# Mood states and well-being of spouses of fibromyalgia patients: a systematic review and meta-analysis

**DOI:** 10.3389/fpsyg.2024.1411709

**Published:** 2024-09-13

**Authors:** Yulia Treister-Goltzman, Roni Peleg

**Affiliations:** ^1^Department of Family Medicine and Siaal Research Center for Family Practice and Primary Care, The Haim Doron Division of Community Health, Faculty of Health Sciences, Ben-Gurion University of the Negev, Beer-Sheva, Israel; ^2^Clalit Health Services, Southern District, Beer-Sheva, Israel

**Keywords:** fibromyalgia, spouses, caregivers, anxiety, mood disorders, quality of life

## Abstract

**Background:**

We carried out a systematic review of the medical literature on potential effects of caregiving on the health and well being of spouses of Fibromyalgia (FM) patients and pooled the results in a meta-analysis.

**Methods:**

The review is comprised of original studies that examined the mood states and well-being of husbands/wives, or long-term intimate partners, of FM patients. The authors searched the PubMed, Scopus, APA PsycNet and Web of Science databases using the key words “fibromyalgia and spouses,” “fibromyalgia and partners,” and “fibromyalgia and husbands.” Of 570 papers that were initially identified using the search words, 18 papers were considered eligible. We used the Joanna Briggs Institute Critical Appraisal Checklist (JBICAC) and Critical Appraisal Skills Program (CASP) tools to assess the risk of bias in the analytical cross-sectional and qualitative studies, respectively.

**Results:**

The overall score in mood states was significantly higher among spouses of FM patients than among spouses of individuals without FM (SMD [95% CI] = 0.52 [0.30; 0.74]). The strongest evidence was found for depression, SMD [95% CI] = 0.68 [0.33; 1.03]. The overall standardized score of quality of life was significantly lower among spouses of FM patients, SMD [95% CI] = −0.59 [−0.79; −0.38], with significant differences in physical function and role, emotional role, and mental health subscales.

**Limitation:**

Limitation of this review is the scant number of studies that addressed several health domains, which made it impossible to carry out meta-analyses in these domains.

**Conclusion:**

Spouses of FM patients show the emotional and physical consequences of caregiving, and impaired quality of life. Addressing these problems can prevent deterioration of their health and improve their quality of life.

## Introduction

Although informal caregivers can gain satisfaction from their role (Garcia-Mochon et al., 2019), they may also experience negative physical (poor self-rated health, chronic diseases, and functional limitations) and psychological (depression and anxiety) consequences from the caregiving burden (Dong et al., 2019; Monahan et al., 2023; Polenick et al., 2020).

Spousal caregivers report a much higher burden, a lower quality of life, and unique needs compared to caregivers who are adult children, or significant others (Dang et al., 2022; Eom et al., 2017). The higher level of distress among spouses is explained mainly by more time spent with the patient, the higher level of care provision, and lower probability of asking for help (Ahmad et al., 2023). Most studies on the health and wellbeing consequences of caregiving were carried out among caregivers of patients with dementia and cancer.

Fibromyalgia (FM) is a multifactorial syndrome characterized by chronic widespread pain. Although pain is the main symptom among patients with FM, they may also experience functional and cognitive disorders, including fatigue, sleep and mood disorders, and cognitive impairment (Clauw et al., 2023). FM is the third most common musculoskeletal condition in terms of prevalence, after lumbar pain and osteoarthritis (Sarzi-Puttini et al., 2020). Its prevalence peaks at 50–60 years and is estimated at 2–4% (Sarzi-Puttini et al., 2020). It affects women predominantly and may be associated with other conditions, including chronic fatigue syndrome, anxiety, depression, irritable bowel syndrome, and most musculoskeletal rheumatic diseases (Sarzi-Puttini et al., 2020). People with FM are frequent utilizers of health care, like patients with diabetes mellitus and hypertension (Bair and Krebs, 2020). They report alarming levels of suicidal ideation (Varallo et al., 2024) and have increased suicide-related mortality (Treister-Goltzman and Peleg, 2023). The combination of high prevalence, involvement of patients in their productive years, altering physical and mental health, and economic burden caused by the loss of productivity and high health services utilization turns FM into a serious public problem.

The FM-related disability rate is high, averaging 35% throughout the world and is continuously increasing (Ben-Yosef et al., 2020). The mechanism for altering function in FM patients is complex and involves both physical and mental symptoms. Frequent psychiatric and rheumatic comorbidities aggravate the situation (Ben-Yosef et al., 2020). The onset of FM is usually at a relatively young age and the disease has a chronic course, so spouses of FM patients spend many years providing physical help, and emotional support. The pain and functional impairment caused by the disease may have a negative effect on the patient’s family members, especially spouses, and impact their mood states and well-being.

*Mood* or mental–emotional states reflect feelings and are commonly used to measure the state of mind and general stress on patients (Petrowski et al., 2021). Anxiety and Depression are the most prevalent mental–emotional disorders and often coexist. They and other mood disturbances are frequently grouped for research purposes into six dimensions: Tension-Anxiety, Depression-Dejection, Anger-Hostility, Vigor-Activity, Fatigue-Inertia, and Confusion-Bewilderment (McNair et al., 1971; López-Jiménez et al., 2021). Mood states are assessed by individual measurement tools (such as the Beck Anxiety Inventory or the Hospital Anxiety Depression scale for Anxiety), or by a composite measure, such as the Profile of Mood States.

*Well-being* is a broad conceptual definition that is used across disciplines to portray a state of wellness, health, satisfaction, and happiness. It is a multifaceted construct that encompasses quality of life, economic, emotional, physical, sexual, and spiritual dimensions (Bautista et al., 2023). Each of these dimensions can be assessed by a dedicated scale or by a qualitative interview.

The aim of this study was to carry out a systematic review of the medical literature on potential effects of cohabitation and caregiving on the mood states and well-being of spouses of FM patients and to pool the results in a meta-analysis.

## Methods

### Search strategy for identification of studies

The electronic databases PubMed, Scopus, APA PsycNet, and Web of Science were searched systematically over the month of December 2023 to identify studies, from any date and in any language, that are related to the effects on the health and well being of caregiving on spouses of FM patients. The search was updated in July 2024. We followed the MOOSE Reporting Guidelines for Meta-analyses of Observational Studies. Prior to performing the review, it was registered at the PROSPERO registration site (registration # CRD42023485272).

The search was conducted using three different combinations of keywords: “fibromyalgia and spouses,” “fibromyalgia and partners,” and “fibromyalgia and husbands,” in each database. Only spouses of patients with physician diagnosed, not self-reported FM, were included. Since in real life clinical practice, FM patients are a mixed population, diagnosed at different times by different criteria that were relevant at the time of diagnosis, we didn’t limit the review by the criteria with which FM was diagnosed (American College of Rheumatology criteria of 1990, 2010, or 2016).

In order to obtain a full picture of the effects of caregiving on spouses of FM, the review included qualitative and quantitative studies, studies that included a comparison group, and those that didn’t. The inclusion criteria were: (1) the study was original research, (2) it evaluated the effects of cohabitation and caregiving on the mood states and well-being of spouses of FM patients, (3) studies that examined mood states addressed anxiety, or depression, or one of the dimensions listed in the definition of the mood states above; if the study related to well-being, it included one of the dimensions described in the definition of well-being above, and (4) the spouses of FM patients were either husbands/wives or long-term intimate partners. The exclusion criteria were: (1) the study was not original research (review articles, case reports, book chapters), (2) FM was self-reported and not diagnosed by a physician, (3) the health implications related to purely physical illness, e.g., hypertension or diabetes, and (4) the spouses were interviewed on different health and well-being aspects of FM patients, but not its effects on them.

In cases where the study population was a mixed population of informal caregivers, and not only spouses, it was included in the review only if it was possible to extract data on spouses separately. In cases, in which the study focused on the health and well-being of both FM patients and their spouses, it was included and only the relevant data on spouse’s health effects were extracted.

In the first phase, all the abstracts were evaluated for inclusion and exclusion criteria. This phase was carried out by a single investigator (YTG). In the second phase, both investigators read the full texts of the selected abstracts chosen in the first phase and conducted a comprehensive, independent review of all the papers and their bibliographies to identify additional potentially relevant papers. In cases of disagreement the paper was discussed until a joint decision was reached.

### Data collection

The following data were collected: author and year of publication, study type, number of participants, percent of male spouses, mean age, duration of marriage/cohabiting with FM patients, years since FM diagnosis, years since the beginning of symptoms (where possible), main outcome measures (aspects of health and well-being of spouses that were assessed) and findings.

### Assessment of risk of bias

The assessment of risk of bias was carried out separately for quantitative and qualitative studies. For quantitative studies we used the Joanna Briggs Institute Critical Appraisal Checklist (JBICAC) for analytical cross-sectional studies (Moola et al., 2020). It was developed by an international research organisation based in the Faculty of Health and Medical Sciences at the University of Adelaide, South Australia and is a recommended and widely used tool for assessing the quality of analytical cross-sectional studies (Ma et al., 2020). Bias domains included in this tool are (1) criteria for inclusion in the sample, (2) description of study subjects and the setting, (3) measurement of the exposure, (4) criteria for measurement of the condition, (5) identifying confounding factors, (6) strategies to deal with confounding factors, (7) measurement of the outcomes, (8) use of appropriate statistical analysis (Moola et al., 2020). The answer to the question that assesses every domain can be “yes,” “no,” “unclear” or “not applicable.” The overall appraisal of the study varies from “include,” to “exclude” and “seek further evidence.” For the second, qualitative studies group, the risk of bias was assessed by the Critical Appraisal Skills Program (CASP) tool. This checklist was designed to be used as an educational pedagogic tool and does not incorporate a scoring system. It is based on JAMA “Users’ guides to the medical literature 1994″ and piloted with health care practitioners (Critical Appraisal Skills Programmme, 2018). CASP includes six questions exploring the validity of the study (aims, methodology and recruitment strategy), three questions on ethical issues and data analysis, and one question on contribution to existing knowledge (Critical Appraisal Skills Programmme, 2018). The answer to the questions can be: “yes,” “no” or “cannot tell,” and together they help to assess the quality of the research. Nowadays CASP is the most frequently recommended tool for assessing qualitative studies (Ma et al., 2020).

### Data synthesis and analysis

The findings of the quantitative studies were grouped and described according to the type of assessed health outcome, e.g., mood state, quality of life, etc. Meta-analyses were performed if more than two studies assessed the same outcome. Meta-analyses were performed using the inverse-variance method with Metafor, Meta, and Demtar packages for R software (version 4.3.1) (R Core Team, 2021). As we anticipated considerable between-study heterogeneity, a random-effect model was used to pool effect sizes. Since different studies used different scales to assess the outcomes, standardized mean differences (SMD) in outcomes were calculated between Fibromyalgia spouses and the comparison group. The sign of differences was reversed, where appropriate, to assure that all studies had the same scale direction. SMD was computed using Hedge’s g statistic (Harrer et al., 2021) with cutoffs 0.2, 0.5, and 0.8 interpreted as small, medium and large effects, respectively. Heterogeneity across the studies was assessed using the I^2^ (inconsistency index) measure to describe the percentage of the variability of the effect due to heterogeneity. A value above 50% or *p* < 0.1 indicated statistically significant heterogeneity (Harrer et al., 2021). Themes that arose in qualitative studies were summarized and described.

## Results

**Figure 1:**
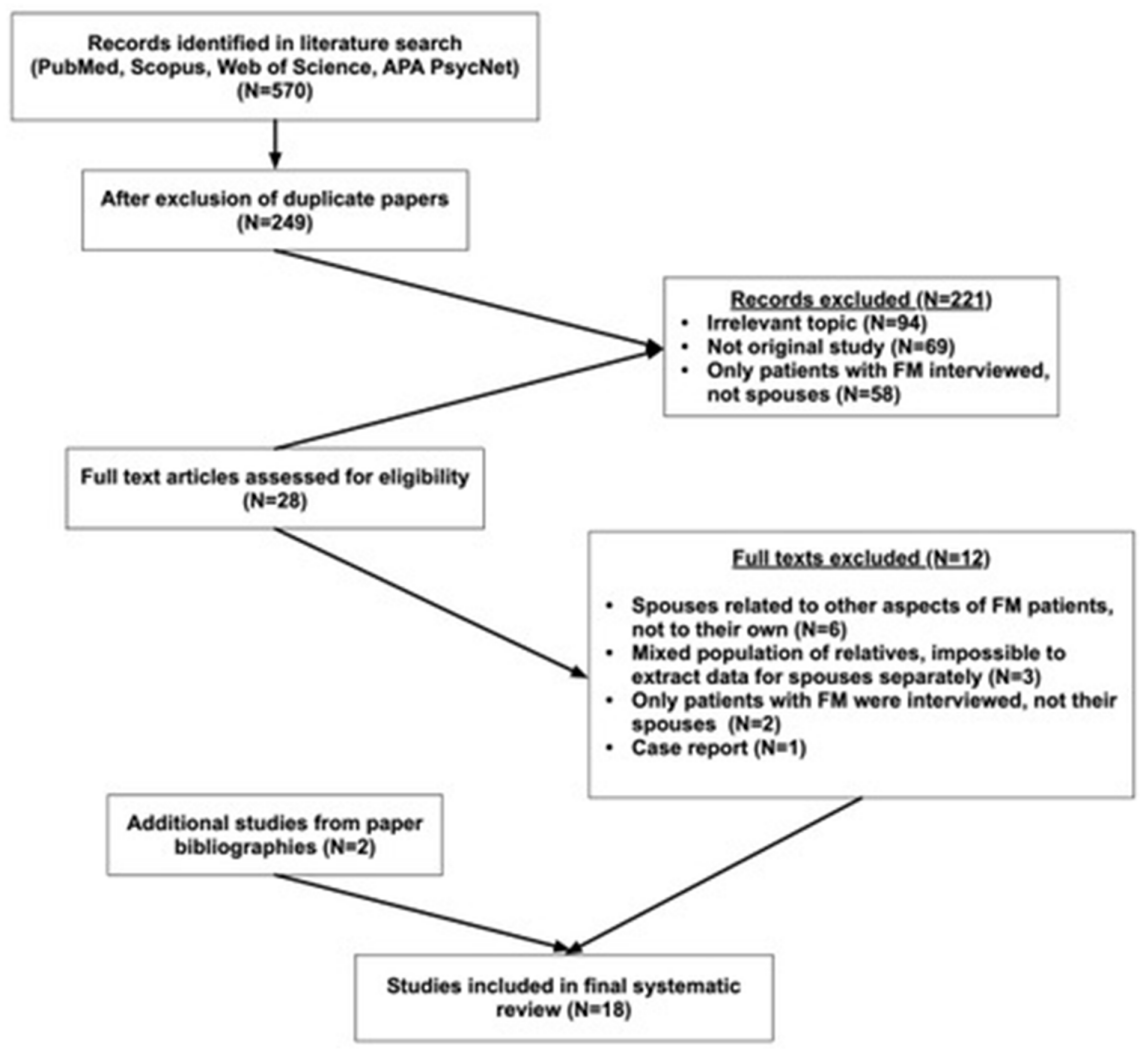
Flowchart of review process.

Of 570 articles identified through the literature search, eighteen studies on the potential effects of cohabitation and caregiving on the health and well being of spouses of FM patients were included in the systematic review (Figure 1). Two hundred and twenty-one papers were excluded during the abstract search phase, as it was clear from the titles or the abstracts that the papers had irrelevant topics, the study was not an original study, or study participants were FM patients, not their spouses (94, 69, and 58 papers, respectively). Twenty-eight full text articles were assessed for eligibility. In six of these the spouses of FM patients related to the feelings of FM patients, not their own, in three of them a mixed population of relatives was interviewed, in two of them only FM patients, not spouses, were interviewed, and one was a case report. Ten of the included studies were cross-sectional analytical (Bigatti and Cronan, 2002; Bigatti et al., 2008; Celepkolu et al., 2021; Collazo et al., 2014; Dewan et al., 2024; Grafft and Lyons, 2024; Parlak et al., 2022; Steiner et al., 2010; Tutoglu et al., 2014; Yener et al., 2015), and eight qualitative (Macdedo et al., 2015; Monteso-Curto et al., 2022; Paulson et al., 2003; Rodham et al., 2010; Romero-Alcala et al., 2019; Soderberg et al., 2003; Sylvain and Talbot, 2002; Vázquez Canales et al., 2024). Two studies were published in Spanish (Collazo et al., 2014; Macdedo et al., 2015), and the rest in English. Articles published in Spanish were translated jointly by two native Spanish speaking physicians.

There were 1,629 participants in the quantitative studies, of them 1,065 spouses of the patients with FM. The rest comprised the comparison groups. Fifty-eight spouses of fibromyalgia patients were interviewed in qualitative studies. The mean age of participants in the quantitative studies ranged from 35.5–59.0 years. The range of age in qualitative studies varied from 35 to 71 years. The studies included participants from the United States, Canada, Sweden, England, Spain, Brazil and Turkey. They related to different aspects of physical, mental, and social health and well-being of the spouses of FM patients. Only 36 of the participants were the wives of patients with FM, the rest were husbands. A summary of studies on the health effects of spouses/partners of FM patients is presented in Table 1.

**Table 1:** Studies included in the systematic review on effects of caregiving on the health and well being of spouses of FM patients.

Study	Design	Country	Participants	Male spouses (%)	Age, years, mean (SD)/median (range)	Duration of cohabiting, years, mean (SD)	Years since FM diagnosis/beginning of symptoms, mean (SD)	Main outcome measures	Findings
**a. Quantitative studies**									
Bigatti et al. (2002)	Analytical cross-sectional, including comparison group	USA	135 husbands of FM patients and 153 husbands of women without FM	100	59 (11)	27 (15)	9.0 (13.0) (since diagnosis)	Health status, mood state, subjective stress, and quality of life	Poorer health and mood states, higher depression, and subjective stress among husbands of FM patients
Bigatti et al. (2008)	Analytical cross-sectional	USA	135 husbands of FM patients	100	59 (11)	27 (15)	9.0 (13.0) (since diagnosis)	Caregiving responsibilities, role strain, association with mood, and mediation by social support and coping	High caregiving responsibilities, which were associated with role strain. Total role strain was related to mood. Social support and emotion-based coping partially mediated this relation.
Steiner et al. (2010)	Analytical cross-sectional, including comparison group	USA	135 husbands of FM patients and 153 husbands of women without FM	100	59 (11)	27 (15)	9.0 (13.0) (since diagnosis)	Marital satisfaction, its association with role strain and mediation by social support and coping (problem based vs. emotional)	Lower marital satisfaction and higher role strain among husbands of women with FM. Social support alone mediated the relationship between role strain and marital satisfaction.
Tutoglu et al. (2014)	Analytical cross-sectional, including comparison group	Turkey	32 husbands of FM patients and 30 husbands of women without FM	100	35.5 (4.3)	ND	2.5 (3.1) (since diagnosis)	Anxiety, depression, sexual function, and quality of life	Higher depression and erectile dysfunction scores, and lower quality of life among husbands of FM patients
Collazo et al. (2014)	Analytical cross-sectional, including comparison group	Spain	60 spouses of FM patients and 60 spouses of women without FM	100	51.7 (9.7)	26.2 (10.2)	3.8 (3.5) (since diagnosis); 12.1 (9.2) (since symptoms)	Personality traits among spouses of FM patients	Personality disorders were more common among spouses of FM patients. Severity of FM and years of cohabitation were positively associated with the probability of personality disorders.
Yener et al. (2015)	Analytical cross-sectional, including comparison group	Turkey	30 spouses of FM patients and 30 spouses of women without FM	100	ND	ND	ND	Anxiety and depression	Higher anxiety and depression scores among spouses of FM patients
Celepkolu et al. (2021)	Analytical cross-sectional, including comparison group	Turkey	30 spouses of FM patients and 38 spouses of women without FM	100	45.0 (10.7)	ND	ND	Anxiety, depression, quality of life, and quality of sleep	Higher anxiety and depression scores and poorer quality of life among spouses of FM patients
Parlak et al. (2022)	Analytical cross-sectional, included comparison group	Turkey	100 spouses of FM patients and 100 spouses of women without FM	100	45.9 (8.6)	21.1 (9.1)	ND	Anxiety, depression, chronic fatigue, quality of life and quality of sleep	Higher anxiety, depression and chronic fatigue scores and poorer quality of life and sleep among spouses of FM patients
Dewan et al. (2024)	Analytical cross-sectional	USA	204 spouses of FM patients	95	57.5 (12.5)	28.2 (16.0)	12.7 (8.2)	Effect of affectionate behavior and communication on mental quality of life	Communication problems, but not affectionate behavior, were associated significantly with mental quality of life
Grafft et al. (2024)	Analytical cross-sectional	USA	204 spouses of FM patients	95	57.5 (12.5)	28.2 (16.0)	12.7 (8.2)	Association of incongruence of pain perception by FM patients and partners with depression and anxiety	Perception of pain severity, but not incongruence between partners, was associated with depression and anxiety among spouses of FM patients
**b. Qualitative studies**									
Sylvain et al. (2002)	Qualitative, through in-depth interviews and group meetings	Canada	Four husbands of FM patients	100	53 (47–65)	28 (21–43)	4 (1–10) (since diagnosis); 12 (4–20) (since symptoms)	Impact of FM on daily life	Felt neglected, lack of support
Paulson et al. (2003)	Qualitative, through in-depth narrative interviews	Sweden	14 wives of FM patients	0	35–54		6–26 (since diagnosis)	Impact of FM on daily life	Struggling to keep going, feeling exhausted, lack of understanding and support, escaping to work.
Soderberg et al. (2003)	Qualitative, through in-depth narrative interviews	Sweden	Five husbands of FM patients	100	50–60	25–40	ND	Impact of FM on daily life, relationships with children and others	Increasing responsibility and workload at home, changing relationships with spouses, friends, and relatives, deepening relationships with children
Rodham et al. (2010)	Qualitative, through in-depth narrative interviews	England	Four husbands of FM patients	100	38–59	5–33	3–15 (since diagnosis), 6–25 (since symptoms)	Impact of FM on daily life	Increasing responsibility and workload at home, changing relationships with spouses and friends
Macdedo et al. (2015)	Qualitative. Individual narrative and semi-structured interviews	Brazil	Four husbands of FM patients	100	41–59	18–32	2–14 (since diagnosis), 4–29 (since symptoms)	Impact of FM on daily life and sexuality	Increased and redistributed workload at home, exhaustion. Negative influence on sexual life.
Romero-Alcala et al. (2019)	Qualitative, through focus groups and in-depth narrative interviews	Spain	18 male partners of FM patients	100	50.1 (8.7), (37–58)	23.1 (13.1), (6–53)	3.7 (2.8), (1–10) (since diagnosis)	Impact of FM on sexual life and sexuality	Coping with new sexuality, resisting the loss of the sexuality
Monteso-Curto et al. (2022)	Qualitative, through focus groups	Spain	Four husbands and one wife of FM patients	80	51–71	ND	14–52 (since diagnosis)	Emotions and coping strategies associated with having a spouse with FM.	Emotional concerns and exhaustion, limitation of leisure activities, escape coping
Vázquez Canales et al. (2024)	Qualitative, through focus groups	Spain	Four husbands and one wife of FM patients	100	42–56	ND	ND	Impact of FM on daily life	Increasing responsibility and workload at home, changing relationships with spouses and friends, limitation of leisure activities, change in sexual life

### Main outcomes of the quantitative studies

#### Studies on mood states of spouses of FM patients

**Figure 2:**
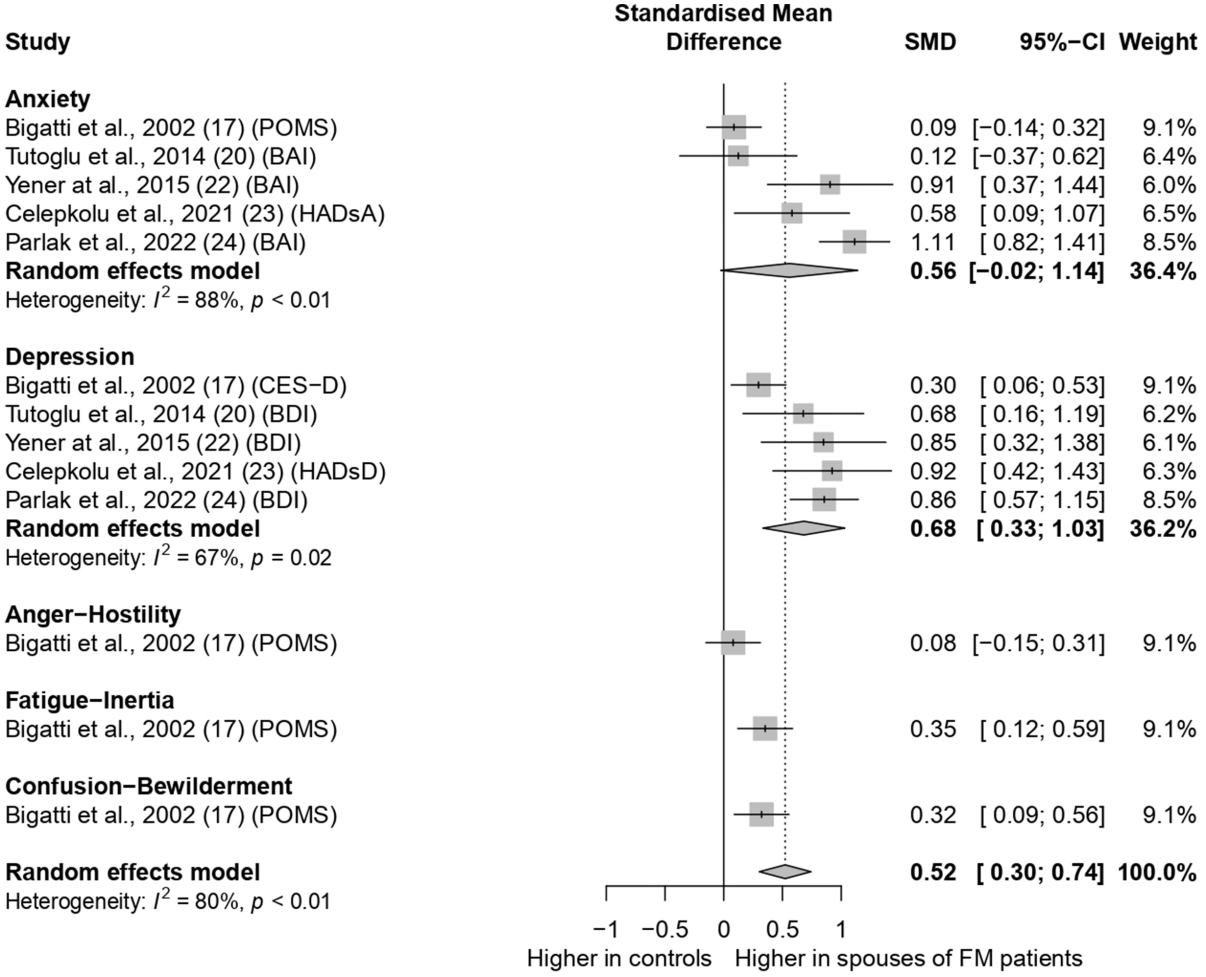
Forest plot on mood states in spouses of Fibromyalgia patients. POMS-profile of mood states, BAI, beck anxiety inventory; HADsA, hospital anxiety depression scale for anxiety; CES-D, center for epidemiological studies depression scale; BDI, beck depression inventory; HADsD, hospital anxiety depression scale for depression.

A Forest plot of the meta-analysis is shown in Figure 2. Five studies (Bigatti and Cronan, 2002; Celepkolu et al., 2021; Parlak et al., 2022; Tutoglu et al., 2014; Yener et al., 2015) compared anxiety among spouses of FM patients and controls. When the results of the studies were pooled, the anxiety score was borderline higher among spouses of FM patients than among spouses of individuals without FM, with SMD [95% CI] = 0.56 [−0.02; 1.14], I^2^ = 88%. The same five studies compared depression scores among spouses of FM patients and controls. In a subgroup meta-analysis, the depression score was significantly higher among spouses of FM patients than among spouses of individuals without FM, with SMD [95% CI] = 0.68 [0.33; 1.03], I^2^ = 67%. One study compared each of the following moods: Anger-Hostility, Fatigue-Inertia and Confusion-Bewilderment (17) with SMDs [95% CI] of 0.08 [−0.15; 0.31], 0.35 [0.12; 0.59], and 0.32 [0.09; 0.56] respectively. The overall score in mood states was significantly higher among spouses of FM patients than among spouses of individuals without FM with a moderate size effect (SMD [95% CI] = 0.52 [0.30; 0.74]). Although the studies were heterogeneous in terms of effect size and statistical significance (I2 = 80%, *p* < 0.01), it’s worth noting that the direction of the effect was similar, i.e., in all studies higher scores of mood disturbances were demonstrated for spouses of FM patients than for spouses of individuals without FM.

#### Studies on quality of the life of spouses of FM patients

**Figure 3:**
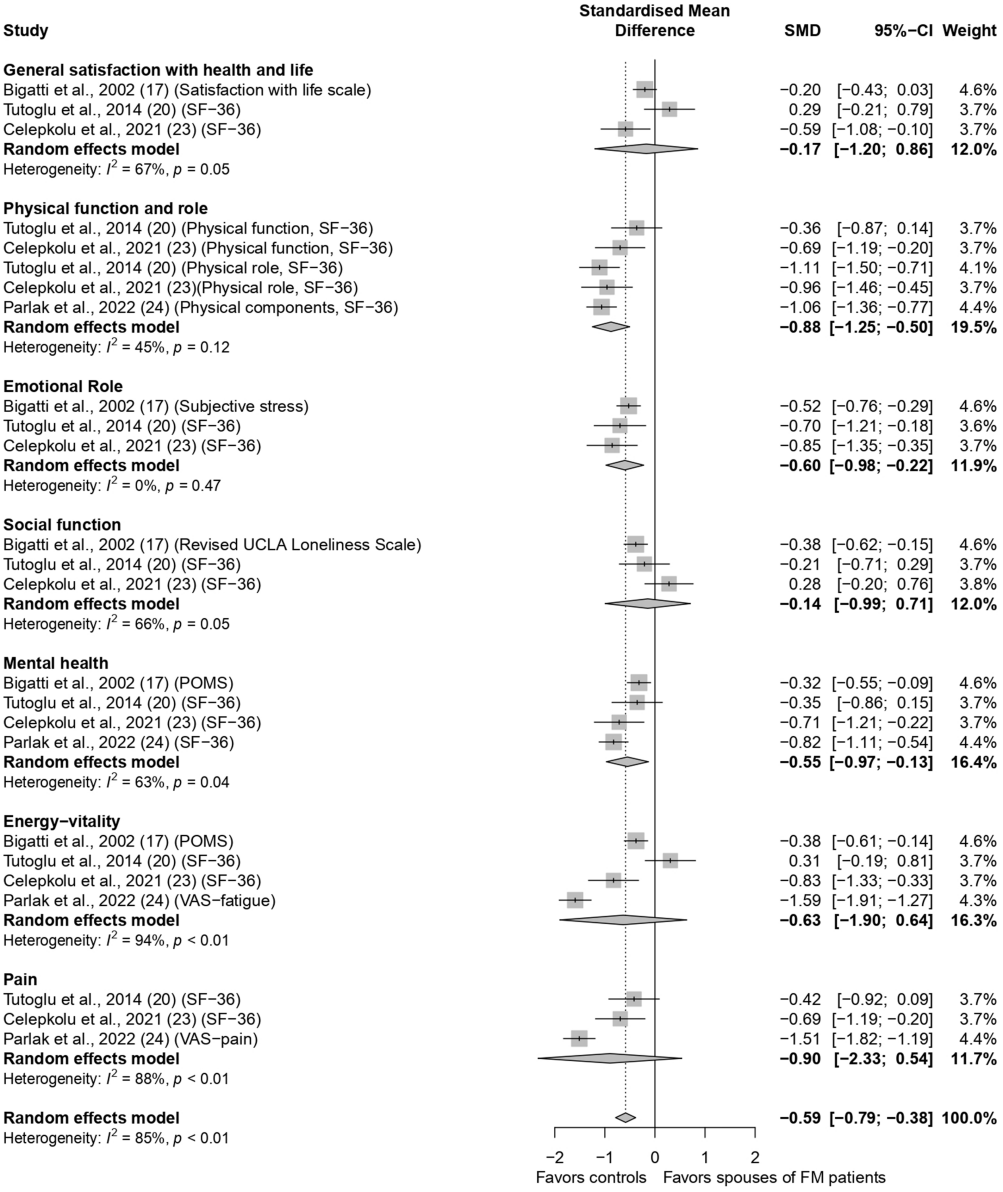
Forest plot on quality of life in spouses of fibromyalgia patients. SF-36, short form-36; POMS, profile of mood states; VAS, visual analogue scale.

Four studies (Bigatti and Cronan, 2002; Celepkolu et al., 2021; Parlak et al., 2022; Tutoglu et al., 2014) compared quality of life among spouses of FM patients and controls. A Forest plot of the meta-analysis is shown in Figure 3. The overall standardized score of quality of life was significantly lower among spouses of FM patients than among spouses of individuals without FM, with moderate sized SMD [95% CI] = −0.59 [−0.79; −0.38], with high heterogeneity (I^2^ = 85%, *p* < 0.01). Although the difference in SMD in some individual subscales did not reach statistical significance, the direction of the effect was negative (lower in spouses of FM patients than in controls) in pooled effects of all subscales. Of note, quality of life was significantly lower in FM spouses’ group than among comparison group in the following subscales: physical function and role (SMDs [95% CI] = −0.88 [−1.25; −0.50], I^2^ = 45%, *p* < 0.01), emotional role (SMDs [95% CI] = −0.60 [−0.98; −0.22], I^2^ = 0%, *p* = 0.47), and mental health (SMDs [95% CI] = −0.55 [−0.97; −0.13], I^2^ = 63%, *p* = 0.04).

#### Main outcomes on other health effects on FM patients’ spouses from quantitative studies

Two studies (Celepkolu et al., 2021; Parlak et al., 2022) compared sleep quality among spouses/partners of patients with FM and comparison groups. In both, the Pittsburgh Sleep Quality Index (PSQI) was used. Lower scores in this tool indicate better sleep. Although in both studies (Celepkolu et al., 2021; Parlak et al., 2022) PSQI was lower in the comparison group than in FM spouses’ group [4.37 ± 2.01 vs. 5.23 ± 2.6 (*p* = 0.123), and 3.58 ± 2.51 vs. 5.1 ± 3.05 (*p* < 0.001), respectively], the difference was statistically significant in one only (Parlak et al., 2022). Another study (Tutoglu et al., 2014) assessed sexual function among spouses of FM patients and found statistically significant lower scores in erectile function, but not in orgasmic function, sexual desire, intercourse satisfaction, and overall satisfaction. The assistance in basic and instrumental activities of daily living (BADL and IADL) that husbands of patients with FM needed to provide to their spouses was assessed in another study (Bigatti et al., 2008), which did not include a comparison group. It showed that FM patients had a greater need for IADL (2.93 ± 0.24) than BADL (1.02 ± 0.13). In studies, that were conducted in the same group of FM spouses (Bigatti et al., 2008; Steiner et al., 2010), the association of role strain with mood (Bigatti et al., 2008), with marital satisfaction (Steiner et al., 2010), and the mediation of these associations by social support and coping (Bigatti et al., 2008; Steiner et al., 2010) were examined. They showed a high role strain in social environment and sexual relation domains, and associations between role strain with mood (Bigatti et al., 2008), and marital satisfaction (Steiner et al., 2010). Social support and emotion-focused coping partially mediated the relation between role strain and mood (Bigatti et al., 2008). Social support alone mediated the relationship between role strain and marital satisfaction (Steiner et al., 2010). A study (Collazo et al., 2014) evaluated the presence of personality traits among spouses of FM compared to spouses of people without FM, and certain personality traits were borderline higher or significantly higher among spouses of FM patients: indecisiveness (*p* = 0.07), insecurity (*p* = 0.07), and instability (*p* = 0.006). The presence of these traits was significantly associated with the severity and duration of FM among their spouses, and the duration of cohabitation/marriage (*p* < 0.001). Dewan et al., 2024, assessed the impact of affectionate behavior, such as touching and kissing, and communication problems on the mental quality of life of 204 couples with FM. The mental quality of life of spouses of FM patients was associated significantly with couple communication but not with affectionate behavior. Another study (Grafft and Lyons, 2024), which was conducted on the same sample, examined the association between within-couple incongruence of pain perception and symptoms of depression and anxiety in FM patients and their spouses. Pain intensity perception, but not incongruence, was associated with anxiety and depression among spouses of patients with FM.

### Main findings of the qualitative studies

Eight qualitative studies focused on the impact of FM on the everyday life and experience of spouses of FM patients (Macdedo et al., 2015; Monteso-Curto et al., 2022; Paulson et al., 2003; Rodham et al., 2010; Romero-Alcala et al., 2019; Soderberg et al., 2003; Sylvain and Talbot, 2002; Vázquez Canales et al., 2024). There were common themes in several of the studies. Spouses reported *increasing responsibility and workload at home*, that stemmed from the functional decline of the FM patients (Macdedo et al., 2015; Rodham et al., 2010; Soderberg et al., 2003; Vázquez Canales et al., 2024), *coping with new sexuality*, as a result of generalized pain and fatigue, and at the same time *resisting loss of sexuality* (Macdedo et al., 2015; Romero-Alcala et al., 2019; Vázquez Canales et al., 2024), *changing leisure activities, changing relationships with relatives and friends,* all resulting in lower ability to socialize (Monteso-Curto et al., 2022; Rodham et al., 2010; Soderberg et al., 2003; Vázquez Canales et al., 2024). Partners talked about *feeling exhausted* (Macdedo et al., 2015; Monteso-Curto et al., 2022; Paulson et al., 2003), and *lack of understanding, and feeling of being neglected* (Paulson et al., 2003; Sylvain and Talbot, 2002). They used *escape coping* (Monteso-Curto et al., 2022; Paulson et al., 2003) and admitted *needing guidance and support* (Paulson et al., 2003; Sylvain and Talbot, 2002).

#### Risk of study bias

The results of the JBICAC tool for risk bias assessment in the quantitative studies are shown in Supplementary Table S1. All ten studies were of good quality, with positive results for most domains of quality assurance. The most problematic domain in several studies was whether objective, standard criteria were used for the measurement of the condition, i.e., some of the studies did not provide details on the exact criteria used to diagnose FM. The answer to this question was unclear. The results of the CASP tool for the risk of bias assessment in the qualitative studies are shown in Supplementary Table S2. All studies were of good quality, with a single problematic domain in seven of the eight studies, i.e., the absence of a description of the relationship between the researcher and the participants.

## Discussion

To our knowledge this is the first systematic review and meta-analysis summarizing the scientific literature on the health effects on the spouses/partners of FM patients. The meta-analysis demonstrated a moderate effect on mood. The strongest evidence was for an increased prevalence of depression, a moderate effect on quality of life with lower scores in physical function and role, an emotional role, and mental health domains. Weaker evidence was found for lower quality of sleep, sexual life, and marital satisfaction, and the need for providing BADL and IADL. A small number of studies focused on this topic.

Increased anxiety and depression scores and lower quality of life were previously described among persons who provide care for their spouses who had functional impairment in BADL and IADL (Czeisler et al., 2023; Yoo et al., 2023; Zhao et al., 2023), as well as for spouses with mental and mood problems (Angermeyer et al., 2006; Walke et al., 2018). FM, as a multifaceted condition, affects the physical, cognitive, and mental functioning of patients, thus necessitating caregiving in several domains. The frustrating nature of FM and the lack of effective treatment for it, lead to high health services utilization, including physicians’ visits and hospitalizations (Treister-Goltzman et al., 2023a,b), which undoubtedly necessitate the support and cooperation of spouses. The medications used in FM are characterized by a high rate of adverse effects. FM patients often misuse opioids as pain relievers (Treister-Goltzman et al., 2023a,b), which can aggravate their physical suffering. Its chronic course and its onset at a young to middle age, turns caregiving into a long-lasting process, implying health and financial effects for the spouse (Koumoutzis et al., 2021).

The findings from qualitative studies can help explain the findings from the quantitative studies. Indeed, the feeling of exhaustion, both physical and emotional, from the increased workload at home, the restricted sexual and social life, along with the perception of lack of understanding and support that repeatedly arose in conversations with the spouses of FM patients in the qualitative studies can explain the mood disturbances, reduced physical and emotional roles, mental health, and overall quality of life found in the quantitative studies. One study found that domestic workload was associated with quality of life only in male caregivers who are, in fact, most FM patients’ spouses (Rico-Blazquez et al., 2022).

The results of the review provide particularly strong evidence for affective disorders in FM patients’ spouses. Both separate meta-analysis on mood disorders and the emotional role subscale of quality of life showed a significantly poorer emotional state in spouses of FM patients, with zero heterogeneity between the studies in the latter. Coping strategies were found to mediate between role strain and mood disturbances in spouses of FM patients (Bigatti et al., 2008). The results of the qualitative studies also pointed to the high prevalence of maladaptive escape coping among spouses with FM patients. This type of coping is considered unhealthy because it often exacerbates stress, creating more anxiety and depression over time (Jun et al., 2019).

Another important issue pinpointed by the qualitative studies in this review is the request and need for guidance and support by the spouses. One study (Steiner et al., 2010) demonstrated that social support alone mediates the association between role strain and marital satisfaction. Multiple studies have demonstrated that after controlling for the caregivers’ sociodemographic and other characteristics, informal social support was significantly associated with lower caregiver burden (McGarrigle et al., 2023; Shiba et al., 2016). Interestingly, in the large study (Shiba et al., 2016), formal social support was associated with lower caregiver burden only if it was provided by the family physicians. This is not surprising since the ongoing follow-up and treatment of patients and their spouses is primarily the responsibility of family physicians. Providing social support, including family intervention, is a part of family residency training worldwide (Korin et al., 2014). Our study focused on FM spouses as a vulnerable group of caregivers and highlighted their need for support and assistance.

Several studies have shown a dyadic association of communication skills with anxiety, depression and emotional QOL in couples living with FM (Dewan et al., 2024; Grafft and Lyons, 2024). To our knowledge no study has focused on the effectivenes of intervention programs on spouses of patients with FM. However, studies that focused on spouses of patients with chronic pain in general, showed that couple-based intervention through increasing social support, paying attention to the neglected needs of caregivers, and promoting patients’ awareness of their spouses’ support can improve the quality of life of both patients and their spouses (Rouhi et al., 2020; Marini et al., 2021). There is a need for studies that assess the effectiveness of similar interventions in couples living with FM.

In addition to the consolidation of existing knowledge on the effects of FM on the mood and well-being of the spouses of FM patients, which could guide physicians and social services to provide care to this population in a more efficient way, the present review outlined the domains for which the evidence is less strong and more research is needed, and suggested new avenues for interventional studies to improve the well-being of the spouses of FM patients.

### Strengths and limitations

The main strength of this systematic review lies in the robust and comprehensive assessment of the study topic, which addressed multiple domains of the health ramifications of cohabitation with FM patients. Additional strengths are the use of multiple databases without limiting the search by date or language of publication, which resulted in inclusion of studies in languages other than English, minimizing the possibility of publication bias. Combining the findings from quantitative and qualitative research completed the picture and contributed to a better understanding of the topic. The main limitation of the review is the scant number of studies that addressed health domains such as sexual life or sleep quality, which made it impossible to carry out meta-analyses in these domains.

### Conclusion

Spouses of FM patients have physical, emotional, and mental health consequences of caregiving, which affect multiple domains of quality of life. They report increased workload, feel exhausted, use maladaptive coping strategies, and speak of lack of understanding and need for support. Given the chronic nature and relatively early onset of FM, the spouses of these patients have long-term caregiving responsibilities, for the most part in their productive years. Addressing the emotional problems and needs of FM patients’ spouses and providing guidance and support can prevent deterioration in their health and quality of life.

## Data Availability

The raw data supporting the conclusions of this article will be made available by the authors, without undue reservation.
