# Supplementary material for: Mood states and well-being of spouses of fibromyalgia patients: a systematic review and meta-analysis
Source: Front Psychol. 2024 Sep 13;15:1411709. doi: 10.3389/fpsyg.2024.1411709 (PMC11427266; doi:10.3389/fpsyg.2024.1411709)
Supplement: Supplementary file 1 [file Table_1.DOCX]

**Supplementary Table 1. The Joanna Briggs Institute (JBI) Critical Appraisal Checklist for analytical cross-sectional studies**

| ***Study*** | ***Were the criteria for inclusion in the sample clearly defined?*** | ***Were the study subjects and the setting described in detail?*** | ***Was the exposure measured in a valid and reliable way?*** | ***Were objective, standard criteria used for measurement of the condition?*** | ***Were confounding factors identified?*** | ***Were strategies to deal with confounding factors stated?*** | ***Were the outcomes measured in a valid and reliable way?*** | ***Was appropriate statistical analysis used?*** | ***Overall appraisal*** |
| --- | --- | --- | --- | --- | --- | --- | --- | --- | --- |
| Bigatti et al., 2002 | Y | Y | Y | U | Y | Y | Y | Y | Included |
| Bigatti et al., 2008 | Y | Y | Y | U | Y | Y | Y | Y | Included |
| Steiner et al., 2010 | Y | Y | Y | U | Y | Y | Y | Y | Included |
| Tutoglu et al., 2014 | Y | Y | U | Y | Y | Y | Y | Y | Included |
| Collazo et al., 2014 | Y | Y | Y | Y | Y | N | Y | Y | Included |
| Yener et al., 2015 | Y | U | Y | Y | U | Y | Y | Y | Included |
| Celepkolu et al., 2021 | Y | U | Y | U | Y | N | Y | Y | Included |
| Parlak et al., 2022 | Y | Y | Y | U | Y | N | Y | Y | Included |
| Dewan et al., 2024, | Y | Y | Y | Y | Y | Y | Y | Y | Included |
| Grafft & Lyons, 2024 | Y | Y | Y | Y | Y | Y | Y | Y | Included |

Y- “yes”, N- “no”, U- “unclear”
