# Supplementary material for: Mood states and well-being of spouses of fibromyalgia patients: a systematic review and meta-analysis
Source: Front Psychol. 2024 Sep 13;15:1411709. doi: 10.3389/fpsyg.2024.1411709 (PMC11427266; doi:10.3389/fpsyg.2024.1411709)
Supplement: Supplementary file 2 [file Table_2.DOCX]

**Supplementary Table 2.** Risk of bias assessment in qualitative studies with Critical Appraisal Skills Programme (CASP)

| **Study** | 1. **Are the results of the study valid?** | | **Is it worth continuing?** | | | | 1. **What are the results?** | | | 1. **Will the results help locally?** |
| --- | --- | --- | --- | --- | --- | --- | --- | --- | --- | --- |
|  | ***Was there a clear statement of the aims of the research?*** | ***Is a qualitative methodology appropriate?*** | ***Was the research design appropriate to address the aims of the research?*** | ***Was the recruitment strategy appropriate to the aims of the research?*** | ***Was the data collected in a way that addressed the research issue?*** | ***Has the relationship between researcher and participants been adequately considered?*** | ***Have ethical issues been taken into consideration?*** | ***Was the data analysis sufficiently rigorous?*** | ***Is there a clear statement of findings?*** | ***How valuable is the research?*** |
| Sylvain et al., 2002 | Y | Y | Y | Y | Y | C | Y | Y | Y | Y |
| Paulson et al., 2003 | Y | Y | Y | Y | Y | Y | Y | Y | Y | Y |
| Soderberg et al., 2003 | Y | Y | Y | Y | Y | C | Y | Y | Y | Y |
| Rodham et al., 2010 | Y | Y | Y | Y | Y | C | Y | Y | Y | Y |
| Macedo et al., 2015 | Y | Y | Y | Y | Y | C | Y | Y | Y | Y |
| Romero-Alcala et al., 2019 | Y | Y | Y | Y | Y | C | Y | Y | Y | Y |
| Monteso-Curto et al., 2022 | Y | Y | Y | Y | Y | C | Y | Y | Y | Y |
| Vázquez Canales et al., 2024 | Y | Y | Y | Y | Y | C | Y | Y | Y | Y |

Y- “yes”, C- “can’t tell
